# Association of Hexachlorobenzene (HCB), Dichlorodiphenyltrichloroethane (DDT), and Dichlorodiphenyldichloroethylene (DDE) with *in Vitro* Fertilization (IVF) Outcomes

**DOI:** 10.1289/ehp.1103696

**Published:** 2011-08-29

**Authors:** Shruthi Mahalingaiah, Stacey A. Missmer, Arnab Maity, Paige L. Williams, John D. Meeker, Katharine Berry, Shelley Ehrlich, Melissa J. Perry, Daniel W. Cramer, Russ Hauser

**Affiliations:** 1 Department of Obstetrics, Gynecology and Reproductive Biology, Brigham and Women’s Hospital and Harvard Medical School, Boston, Massachusetts, USA; 2 Department of Epidemiology, Harvard School of Public Health, Boston, Massachusetts, USA; 3 Channing Laboratory, Department of Medicine, Brigham and Women’s Hospital and Harvard Medical School, Boston, Massachusetts, USA; 4 Department of Statistics, North Carolina State University, Raleigh, North Carolina, USA; 5 Department of Biostatistics, Harvard School of Public Health, Boston, Massachusetts, USA; 6 Department of Environmental Health Sciences, University of Michigan School of Public Health, Ann Arbor, Michigan, USA; 7 Department of Environmental Health, Harvard School of Public Health, Boston, Massachusetts, USA; 8 Department of Environmental and Occupational Health, School of Public Health and Health Services, George Washington University, Washington, DC, USA; 9 Vincent Memorial Obstetrics and Gynecology Service, Andrology Laboratory and *In vitro* Fertilization Unit, Massachusetts General Hospital, Boston, Massachusetts, USA

**Keywords:** assisted reproduction, dichlorodiphenyldichloroethylene, dichlorodiphenyltrichloroethane, hexachlorobenzene, prospective cohort

## Abstract

Background: Hexachlorobenzene (HCB), dichlorodiphenyltrichloroethane (DDT), and dichlorodiphenyldichloroethylene (DDE) are persistent chlorinated pesticides with endocrine activity that may adversely affect the early stages of human reproduction.

Objective: Our goal was to determine the association of serum levels of HCB, DDT, and DDE with implantation failure, chemical pregnancy, and spontaneous abortion in women undergoing *in vitro* fertilization (IVF) from 1994 to 2003.

Methods: Levels of HCB and congeners of DDT and DDE were measured in serum collected during the follicular phase. Multivariable-adjusted statistical models accommodating multiple outcomes and multiple cycles per woman were used to estimate the relation between serum pesticide levels and IVF outcomes.

Results: A total of 720 women with a mean ± SD age 35.4 ± 4.2 years at enrollment contributed 774 IVF cycles. All samples had detectable levels of HCB, DDT, and DDE, with median levels of 0.087 ng/g serum for HCB, 1.12 ng/g serum for total DDT, and 1.04 ng/g serum for *p*,*p*´-DDE. Compared with the lowest quartile (Q1) of HCB, the lipid- and multivariable-adjusted odds ratio (OR) for failed implantation was significantly elevated for those with higher HCB quartiles [Q2–Q4; adjusted ORs: for Q2, 1.71; 95% confidence interval (CI): 1.03, 2.82; for Q3, 2.30; 95% CI: 1.39, 3.81; for Q4, 2.32; 95% CI: 1.38, 3.90] and showed a significantly increasing trend (*p* = 0.001). No statistically significant associations were observed between DDT/DDE and IVF outcomes or between HCB and chemical pregnancy or spontaneous abortion.

Conclusions: Serum HCB concentrations were on average lower than that of the general U.S. population and associated with failed implantation among women undergoing IVF.

Hexachlorobenzene (HCB) and dichlorodiphenyltrichloroethane (DDT) are organochlorines (OCs) used in fungus and pest control. These OCs have demonstrated estrogenic activity in *in vitro* assays and adverse reproductive effects in fish and wildlife (Tiemann 2008). Although HCB and DDT have been banned in the United States, HCB is a by-product of organic chemical production processes and is still produced outside the United States [Agency for Toxic Substances and Disease Registry (ATSDR) 2002b]. DDT continues to be used abroad for the control of mosquito-borne diseases (van den Berg 2009).

The main source of human exposure to these OCs is through dietary ingestion. Generally, HCB and DDT are found in meat, fish, and milk products (Brilhante and Franco 2006; Fontcuberta et al. 2008; Mawussi et al. 2009; Yu et al. 2009). DDT can be found in higher concentrations in foods imported from countries still using DDT (ATSDR 2002a). Because of their chemical stability, bioaccumulation up the food chain, and the ongoing production or use in some countries, HCB, DDT, and dichlorodiphenyldichloroethylene (DDE), the primary metabolite of DDT, continue to be detected in human blood (ATSDR 2002a), breast milk (Tsang et al. 2011), follicular fluid (Meeker et al. 2009), amniotic fluid (Foster et al. 2000; Luzardo et al. 2009), and human umbilical cord blood (Jimenez Torres et al. 2006).

There are conflicting data regarding the effect of exposure to DDT/DDE on early reproductive outcomes. A recent study described no association between oocyte, fertilization, and implantation parameters in women undergoing *in vitro* fertilization (IVF) and exposure to DDT (Al-Saleh et al. 2009). Another study found an association between increased time to pregnancy and self-report of exposure to agricultural and home pesticides (Harley et al. 2008). Some studies suggest an association of DDT/DDE with early clinically detected fetal loss (Longnecker et al. 2005; Venners et al. 2005). Other studies reported early pregnancy loss and early neonatal and childhood mortality after a widespread accidental exposure to HCB in Turkey (Jarrell et al. 1998; Peters et al. 1982). Because of concerns raised by these earlier studies, we explored the association of serum levels of these persistent OCs with early reproductive failure uniquely observable within the context of an IVF study.

## Methods

*Study population.* This study population is a nested case–control subset of patients derived from a prospective cohort designed to assess predictors of IVF outcomes. Between August 1994 and June 2003, couples commencing assisted reproduction therapy were enrolled through three Boston, Massachusetts, area clinics. Couples agreeing to participate in this study underwent an informed consent process and provided written consent before enrollment. There were two phases of patient enrollment, 1994–1999 and 2000–2003, corresponding to initial funding and renewal phases. Study protocols were approved by the Institutional Review Board at Brigham and Women’s Hospital and the Human Subjects Committee at Harvard School of Public Health. Approximately 65% of couples who were approached agreed to participate in the study. Couples who required donor gametes, a gestational carrier, or gamete/zygote intrafallopian transfer were excluded from the main study. Most couples underwent multiple IVF cycles (up to six), with an average of two cycles per couple.

Because of budget limitations, we were unable to measure serum OC levels on all of the study participants. Therefore, we devised our case and control selection strategy to maximize statistical power, selecting cases based on outcomes of interest. Of the 2,350 couples in the main study, 765 women were selected by the outcome of their first IVF cycle (failed implantation, chemical pregnancy, spontaneous abortion, or live birth). Because the number of available cases varied by failure type, with failed implantation being most frequent, we analyzed serum HCB/DDT/DDE concentrations for a random sample of 200 women with first-cycle implantation failures. Because chemical pregnancies and spontaneous abortions were less frequent, all cycles from the entire cohort with these outcomes were selected. Also included in the analysis were 62 repeated cycles among a subset of 46 women, from a total of 265 cycles from 110 women who were selected for measurement of DDE, DDT, and HCB in a prior analysis of within-woman variability in serum concentrations of OC compounds from the same parent study (Meeker et al. 2009). A corresponding sample of first-cycle live births was selected using stratified sampling based on age category, study center, and study phase. In total, 765 women contributed 827 cycles.

Data abstracted from clinical records were infertility diagnoses, ovarian stimulation protocol (down-regulation or flare), type and total ampules of gonadotropin, use of intracytoplasmic sperm injection (ICSI), day of embryo transfer (ET), number of embryos transferred, and cycle outcomes.

Only women who proceeded to undergo ET of one or more embryos were eligible for the present analysis. When at least one embryo was transferred but without subsequent rise of human chorionic gonadotropin (hCG) beyond 5.0 mIU/mL, the cycle outcome was defined as a failure of implantation. A chemical pregnancy was defined by a post-ET hCG measurement of ≥ 5.0 mIU/mL with no further embryonic development (gestational sac or yolk sac). Clinical pregnancy was determined by ultrasound visualization of a gestational sac or a fetal heartbeat. Among clinically recognized pregnancies, outcomes included spontaneous abortion (fetal demise before 20 weeks of gestation) or live birth of at least one infant. Other outcomes for clinically recognized pregnancy were ectopic pregnancy, molar pregnancy, and stillbirth (fetal demise at ≥ 20 gestational weeks) and were excluded in the present analysis because numbers were small and lacked power for independent investigation.

*Measurement of HCB, DDT, and DDE in serum.* Blood samples were collected from women during the follicular phase of their first IVF/ICSI cycle and subsequent cycles in a nonfasting state immediately before hCG administration. The serum fraction was separated for all blood samples by centrifugation and stored at –80°C. Measurement of the OC pesticides HCB, *p*,*p*´-DDT, *p*,*p*´-DDE, *o*,*p*´-DDT, and *o*,*p*´-DDE was conducted by the Organic Chemistry Analytical Laboratory, Harvard School of Public Health (Boston, MA), using methods described previously (Korrick et al. 2000). Briefly, after liquid–liquid extraction and column chromatography, samples were analyzed by dual gas chromatography with electron capture detection, on two capillary columns of different polarity using two internal standards. Samples were accompanied by the following quality control samples: a procedural blank, matrix spike samples, and a laboratory control sample. Each sample was spiked with two surrogate compounds to monitor the efficiency of the extraction procedure. All final results were reported after subtracting the amount of the analyte measured in the procedural blank associated with the analytic batch. Method detection limits (MDLs) were determined as recommended by the U.S. Environmental Protection Agency (1984).

The wet-weight MDL values for HCB and congeners of DDT and DDE were all < 0.01 ng/g. The OC concentrations were measured at part per billion concentrations. The within-assay variation for all target pesticides ranged from 2% to 5%. The between-assay variation (percent coefficient of variation) for all target pesticides was < 10%.

Serum total cholesterol and triglycerides were measured enzymatically. Total serum lipid content was calculated by Equation 2 of Phillips et al. (1989) and then used for lipid standardization of serum OC concentration.

*Statistical analysis.* Serum OC concentrations in the study population of 765 women were grouped into quartiles (Q1–Q4). Total DDT was defined for this analysis as a sum of *p*,*p*´-DDT, *o*,*p*´-DDT, *p*,*p*´-DDE, and *o*,*p*´-DDE. The relation between IVF outcomes and quartiles of HCB, *p*,*p*´-DDE, and total DDT was explored with multivariable generalized linear regression models, with data structured to accommodate joint models for multiple outcomes and multiple cycles contributed per woman. Whenever a woman was at risk of a cycle failure because of implantation failure, chemical pregnancy, or spontaneous abortion in each cycle, a binary outcome (1 = failure, 0 = not a failure) was recorded. Thus, a woman provided up to three contributions to risk sets for the possible outcomes within each IVF cycle. A logistic regression model was then employed for each early pregnancy outcome, while adjusting for covariates. Interaction terms between OC quartile and failure type were included in the model to allow for a distinct relation between the OC of interest and reproductive failure type. Interaction terms between age and failure type were also included to account for the possibility of differing age effects on the various failure end points. A woman-specific random-effect term, assumed to be Gaussian, was included to account for the correlation among outcomes from different cycles for the same woman. Trend tests were conducted by assigning ordinal integer values to HCB, DDE, and DDT quartiles (0 = Q1 to 3 = Q4).

Potential confounding variables considered in our analyses were maternal age (< 35, 35–37, 38–40, > 40 years), body mass index (BMI), serum lipids, smoking status (never, past, or current), site (of the three participating Boston area clinics), study phase (1994–1998 or 1999–2003), race (Caucasian or other), previous live birth (yes/no), ampules of gonadotropins, protocol (down-regulation or other), ICSI (yes/no), number of embryos transferred, and primary infertility diagnosis. Primary infertility diagnoses were categorized as male factor, ovulatory dysfunction, and other/unexplained. The “other/unexplained” category included Müllerian anomalies and uterine or tubal pathology. Cycles that were missing race, infertility diagnosis, or previous live birth (6% of cycles) were assigned a missing indicator variable and retained in all analyses. Cycles missing any other covariate information were excluded.

The serum OC concentrations were standardized by dividing by the calculated total serum lipid concentration. In addition, we also modeled IVF outcomes in relation to wet-weight serum levels of the specific and adjusted for serum lipids as a covariate in multivariable models, to reduce bias, as suggested by Schisterman et al. (2005).

Data analysis was conducted using SAS software (version 9.1; SAS Institute Inc., Cary, NC). *p*-Values < 0.05 were considered statistically significant.

## Results

Of the 827 IVF cycles evaluated, 774 (94%) had sufficient covariate information to be included in our analysis, representing 720 (94%) of the 765 women. Among the 54 IVF cycles excluded, 6 were missing BMI, 13 were missing lipids, 2 were missing ampules of gonadotropins, and 32 were missing number of embryos transferred. The 720 women had a mean age of 35 years, were primarily Caucasian (90%) and current nonsmokers (94%), and had a mean BMI of 24.2 kg/m^2^ (Table 1). Unexplained infertility (34%) and male factor (35%) accounted for most infertility diagnoses; the remaining women were diagnosed with either tubal factor (18%) or ovulatory dysfunction (13%). Because of the nested case–control sampling design, the oversampling of failure outcomes on a subset of women was not representative of the failure outcome percentages of the main study. There were 541 total cycle failures, of which 490 were included in the analysis and consisted of implantation failure (209), chemical pregnancy (161), and spontaneous abortion (120).

**Table 1:** Characteristics of all women undergoing IVF eligible for inclusion in the analytic population (*n* = 765) and women included in the final model (*n* = 720).

Characteristic	All (765 women, 827 cycles)	Included (720 women, 774 cycles)
Age (years)		35.9 ± 4.21		35.4 ± 4.20
BMI (kg/m^2^)		24.2 ± 5.09		24.2 ± 5.12
Caucasian		693 (90.6)		651 (90.4)
Cigarette smoking status				
Current smoker		50 (7)		40 (6)
Past smoker		223 (29)		214 (30)
Previous live birth		181 (24)		171 (24)
Total gonadotropins (mIU)*a*		34.4 ± 18		34.4 ± 18
Down-regulation stimulation protocol*a*		604 (73)		569 (74)
ICSI*a*		259 (31)		245 (32)
No. of embryos transferred*a*		3.15 ± 1.33		3.16 ± 1.33
Serum lipids (mg/g)		5.12 ± 1.13		5.22 ± 1.17
Infertility diagnosis*b*				
Tubal factor		143 (19)		129 (18)
Ovulatory dysfunction		99 (13)		92 (13)
Male factor		267 (35)		251 (35)
Unexplained		256 (34)		248 (34)
IVF outcome*a*				
Failed implantation		229 (28)		209 (27)
Chemical pregnancy		177 (21)		161 (21)
Spontaneous abortion		124 (15)		120 (16)
Other (ectopic/stillbirth/molar pregnancy)		11 (1)		10 (1)
Live birth		286 (35)		274 (35)
Values are mean ± SD or *n* (%). **a**Cycle-level data. **b**Primary infertility diagnosis.				

Distributions of serum concentrations of HCB, DDT, and DDE are presented as both wet-weight and lipid-standardized concentrations (Table 2). No samples were below the limit of detection. The geometric mean (GM) and median concentration for HCB were 0.088 and 0.087 ng/g serum, respectively, compared with a GM and median of 0.095 and 0.092 ng/g serum in the general U.S. female population in 2003–2004 [Centers for Disease Control and Prevention (CDC) 2009]. Total DDT had the highest concentration (median = 1.12 ng/g serum, range 0.02–31.8 ng/g serum). Most of the total DDT was from *p*,*p*´-DDE contributions, which accounted for > 90% of the sum of all measured DDT/DDE congeners. The GM and median *p*,*p*´-DDE concentrations were, respectively, 1.09 and 1.04 ng/g serum, with a range of 0.007–30.6 ng/g serum, compared with a GM and median of 1.45 and 1.25 ng/g serum in the general female U.S. population (CDC 2009). The GM and median for *p*,*p*´-DDT in the general female U.S. population included levels below the limit of detection (CDC 2009). There was a strong correlation between the concentration of total DDT and *p*,*p*´-DDE (Spearman correlation coefficient = 0.98). Of the 46 women with serum from multiple cycles ultimately included in the final analysis, 29 (63%) remained in the same HCB quartile across cycles after lipid standardization, similar to the 34 (71%) with lipid adjustment in the multivariable model. HCB concentrations between cycles were also strongly correlated (Spearman correlation coefficient = 0.98).

**Table 2:** Distribution of serum HCB, *p*,*p*´-DDE, and total DDT concentrations among 765 women undergoing IVF.

OC	GM	Minimum	Q1 (mean)	Median	Q3 (mean)	Maximum
Wet weight (ng/g serum)												
HCB		0.088		0.005		0.066		0.087		0.114		2.31
*p*,*p*´-DDE		1.09		0.007		0.659		1.04		1.75		30.6
Total DDT*a*		1.22		0.020		0.741		1.12		1.88		31.8
Lipid standardized (ng/g lipid)												
HCB		18		3.39		13.3		17.5		23.0		426
*p*,*p*´-DDE		226		26.1		132		210		362		6,627
Total DDT*a*		251		31.7		148		233		390		6,977
**a**Total DDT includes the sum of *p*,*p*´-DDT, *o*,*p*´-DDT, *p*,*p*´-DDE, and *o*,*p*´-DDE.												

When serum lipids were adjusted as a covariate with other potential confounders, a dose–response relation between increasing HCB concentration quartile and odds ratios (ORs) for failed implantation was observed (Table 3). Compared with women in Q1, the multivariable adjusted ORs for failed implantation were 1.71 for women in Q2 [95% confidence interval (CI): 1.03, 2.82], 2.30 for women in Q3 (95% CI: 1.39, 3.81), and 2.32 for women in Q4 (95% CI: 1.38, 3.90; *p*-value, test for linear trend = 0.001). When lipid-standardized HCB concentrations were used as the dependent variable, a statistically significant linear increase in ORs for implantation failure remained. Although no statistically significant associations were found between serum HCB and chemical pregnancy, spontaneous abortion, or early pregnancy failure, ORs for early pregnancy failure increased with increasing HCB quartiles. Lack of statistical significance may be due to the relatively small numbers of chemical pregnancies and spontaneous abortions.

**Table 3:** Adjusted ORs*^a^* and 95% CIs for cycle outcomes in relation to serum HCB quartiles among 720 women undergoing IVF.

Models with HCB concentrations standardized by serum lipids (g)					Models adjusted for serum lipids (ng/g) as a covariate*b*				
Outcome/HCB quartile	*n*			*n*		
	Women	Cycles	Events	OR (95% CI)	Women	Cycles	Events	OR (95% CI)
Failed implantation												
Q1		189	204	38		Referent		197	209	41		Referent
Q2		181	203	48		1.33 (0.82, 2.15)		188	208	55		1.71 (1.03, 2.82)
Q3		192	204	65		1.81 (1.11, 2.95)		191	208	67		2.30 (1.39, 3.81)
Q4		191	203	75		2.31 (1.42, 3.77)		195	208	71		2.32 (1.38, 3.90)
*p*_trend_						< 0.001						0.001
Chemical pregnancy												
Q1		189	204	47		Referent		197	209	42		Referent
Q2		181	203	41		0.86 (0.52, 1.44)		188	208	45		1.14 (0.70, 1.91)
Q3		192	204	45		1.30 (0.77, 2.18)		191	208	44		1.43 (0.84, 2.44)
Q4		191	203	41		1.07 (0.62, 1.85)		195	208	46		1.35 (0.78, 2.34)
*p*_trend_						0.49						0.20
Spontaneous abortion												
Q1		189	204	32		Referent		197	209	35		Referent
Q2		181	203	34		1.07 (0.59, 1.95)		188	208	32		1.06 (0.57, 1.93)
Q3		192	204	31		1.40 (0.74, 2.66)		191	208	28		1.12 (0.59, 2.13)
Q4		191	203	25		1.08 (0.55, 2.10)		195	208	29		1.23 (0.64, 2.35)
*p*_trend_						0.59						0.52
Early pregnancy failure												
Q1		189	204	118		Referent		197	209	119		Referent
Q2		181	203	123		1.11 (0.73, 1.70)		188	208	134		1.38 (0.89, 2.13)
Q3		192	204	147		1.96 (1.22, 3.14)		191	208	144		1.91 (1.20, 3.02)
Q4		191	203	145		1.81 (1.11, 2.96)		195	208	149		1.98 (1.19, 3.28)
*p*_trend_						0.003						0.003
**a**Joint models for multiple outcomes and multiple cycles per woman were adjusted for serum lipids, study site, study phase, race/ethnicity, previous live birth, maternal age, BMI, smoking status, ampules of gonadotropins, protocol, ICSI, number of embryos transferred, and primary infertility diagnosis. **b**Wet-weight serum (ng/g serum) measurements were used to assign quartiles, followed by lipid adjustment (g lipid/g serum) using lipid as a covariate in the model; 66% of the cycles had a lipid-adjusted HCB value in the same quartile as the lipid-standardized HCB value.												

Although no statistically significant associations were found between *p*,*p*´-DDE and any individual or combined early reproductive failures, the OR for any of the three early pregnancy failure types among cycles in Q4 *p*,*p*´-DDE versus Q1 was 1.6 (95% CI: 0.98, 2.63) (Table 4). The OR for Q4 total DDT, after lipid adjustment, and early pregnancy failure versus Q1 was 1.85 (95% CI: 1.13, 3.03; *p*-value, test for linear trend = 0.049; data not shown).

**Table 4:** Adjusted ORs*^a^* and 95% CIs for cycle outcomes in relation to serum *p*,*p*´-DDE quartiles among 720 women undergoing IVF.

Outcome/DDE quartile	Models with *p*,*p*´-DDE concentrations standardized by serum lipids (g)					Models adjusted for serum lipids (ng/g) as a covariate*b*				
	*n*			*n*		
	Women	Cycles	Events	OR (95% CI)	Women	Cycles	Events	OR (95% CI)
Failed implantation												
Q1		188	204	48		Referent		195	209	54		Referent
Q2		184	203	52		0.98 (0.61, 1.56)		187	208	56		1.16 (0.72, 1.86)
Q3		187	204	65		1.17 (0.73, 1.86)		193	208	61		1.12 (0.70, 1.81)
Q4		194	203	61		1.14 (0.70, 1.83)		196	208	63		1.19 (0.73, 1.94)
*p*_trend_						0.29						0.56
Chemical pregnancy												
Q1		188	204	43		Referent		195	209	43		Referent
Q2		184	203	45		1.19 (0.72, 1.98)		187	208	40		1.09 (0.64, 1.87)
Q3		187	204	41		1.16 (0.68, 1.96)		193	208	45		1.14 (0.67, 1.81)
Q4		194	203	45		1.19 (0.70, 2.03)		196	208	49		1.36 (0.78, 1.94)
*p*_trend_						0.56						0.30
Spontaneous abortion												
Q1		188	204	30		Referent		195	209	30		Referent
Q2		184	203	32		1.21 (0.66, 2.22)		187	208	32		1.02 (0.55, 1.93)
Q3		187	204	22		0.62 (0.33, 1.18)		193	208	24		0.67 (0.34, 1.30)
Q4		194	203	38		1.40 (0.74, 2.65)		196	208	38		1.33 (0.69, 2.56)
*p*_trend_						0.67						0.70
Early pregnancy failure												
Q1		188	204	124		Referent		195	209	130		Referent
Q2		184	203	131		1.16 (0.76, 1.79)		187	208	129		1.08 (0.70, 1.65)
Q3		187	204	129		0.96 (0.62, 1.47)		193	208	133		1.03 (0.67, 1.59)
Q4		194	203	149		1.52 (0.94, 2.44)		196	208	154		1.61 (0.98, 2.63)
*p*_trend_						0.12						0.09
**a**Joint models for multiple outcomes and multiple cycles per woman were adjusted for serum lipids, study site, study phase, race/ethnicity, previous live birth, maternal age, BMI, smoking status, ampules of gonadotropins, protocol, ICSI, number of embryos transferred, and primary infertility diagnosis. **b**Wet-weight serum (ng/g serum) measurements were used to assign quartiles, followed by lipid adjustment (g lipid/g serum) using lipid as a covariate in the model.												

We additionally evaluated the relations of serum wet-weight concentrations of DDE, DDT, and HCB, without lipid adjustment or lipid standardization, and outcomes, and effect estimates were altered negligibly (data not shown).

## Discussion

Among the 765 women undergoing IVF in this study, all had detectable serum levels of HCB, DDT, and DDE. Implantation failure ORs exceeded 2 for women with serum concentrations of HCB in Q4, compared with women in the lowest quartile. Previous studies within this cohort have shown that serum OC concentrations were strongly correlated with follicular fluid concentrations (*r*-values: HCB, 0.66; *p*,*p*´-DDE, 0.92; *p*,*p*´-DDT, 0.74) (Meeker et al. 2009), and in other populations OCs were detectable in amniotic fluid (Foster et al. 2000), suggesting the possibility of direct gamete and embryo exposure.

We are not aware of another study that describes the association between clinically observed implantation failure and exposure to HCB; therefore, it will be critical that further studies by other investigators replicate this finding. However, a nonhuman primate study demonstrated that HCB exposure induced a dose-dependent suppression of luteal serum progesterone (Foster et al. 1992). Suppressed luteal progesterone may increase the likelihood of implantation failure due to reduced endometrial maturation or of early pregnancy loss from inadequately low levels of progesterone production (Jones and Wentz 1976). Other studies demonstrated that HCB exposure was associated with degenerative changes in ovarian structure, including germ cell destruction in rhesus monkeys (Iatropoulos et al. 1976) and primordial germ cell loss in cynomolgus monkeys (Jarrell et al. 1993), suggesting other potential mechanism of impaired reproductive outcome.

Our study did not find a statistically significant association between HCB and chemical pregnancy or spontaneous abortion. However, exposure to HCB-contaminated foods in Turkey between 1955 and 1957 resulted in detectable HCB in serum drawn 40 years later. In that population, higher HCB levels were associated with a self-reported history of spontaneous abortion (Jarrell et al. 1998). Furthermore, time to pregnancy, a possible surrogate marker for implantation failure, was not assessed in that study.

In the present study, we also found no significant association between serum DDT/DDE and implantation failure, chemical pregnancy, or early pregnancy loss. However, we did observe a nonsignificant OR of 1.6 for any of the three early pregnancy failure types among cycles in *p*,*p*´-DDE Q4 compared with those in Q1.

One study that evaluated serum DDE levels among 1,717 women and assessed past pregnancy outcomes via survey found a significant adjusted OR of 1.4 (95% CI: 1.1, 1.6) for fetal loss in women per 60-µg/L increase of serum DDE (Longnecker et al. 2005). The primary limitation of that study was the retrospective design in which current serum DDE concentrations were associated with past fetal loss. Another study that followed newly married female textile workers in China also found an association between serum total DDT (range, 5.5–113.3 ng/g serum; median, 27.9 ng/g) and subsequent early fetal loss, defined as < 6 weeks of gestation as measured through decline in urinary hCG (Venners et al. 2005). In a smaller study with 99 women undergoing their first cycle of IVF, no statistically significant relationships between serum and follicular concentrations of DDT/DDE and oocyte number, quality, fertilization rates, or pregnancy rates were reported, although serum DDT/DDE concentrations were higher than in our study population. The authors suggested that some “statistically suspicious” associations were observed; however, ORs, CIs, and *p*-values were not given (Jirsova et al. 2010).

One limitation of the present study is that the study population consisted of couples undergoing IVF. It is unknown whether infertile couples respond differently to OC exposure relative to fertile couples or women with untested fertility. Therefore, although the results of this study are internally valid, it is unclear whether the associations of between HCB or DDE/DDT and early pregnancy outcomes would differ among women who have never experienced infertility. However, in the spontaneous conception setting, it would not be possible to evaluate the association between these OC exposures and implantation failure because it is currently unobservable. The second limitation is that serum concentrations were available only for females and not their male partners. However, exposures to certain ubiquitous chemicals have been shown to be correlated within couples undergoing IVF (Mahalingaiah et al. 2008). Certain predictors of serum OC concentration were not available for this analysis, such as past breast-feeding history and country of origin. However, most patients were likely to be of American origin; other known predictors of persistent pesticide exposure were included in our models, including age and smoking.

Finally, the serum sample from which OC concentration and lipid content were quantified was not a fasting sample. Although both lipid content and serum wet weight of OC increase after a meal, they do not increase proportionally. Furthermore, the method chosen for lipid correction does affect the final corrected concentration. The equation used in our study to calculate total lipids was chosen from those suggested by Phillips et al. (1989) and accounts for total cholesterol and triglycerides but does not diminish the differences between fasting and nonfasting lipid profiles. However, when lipids are accounted for, there are data to suggest that there still is a high degree of correlation between a fasting and nonfasting serum sample (0.96) (Longnecker et al. 1996).

Our study had a number of strengths. The prospective cohort from which this nested case–control study population was drawn employed risk set sampling that allowed for collection of questionnaire data and biological samples before the cycle outcome and temporally close to the clinically and biologically relevant outcomes of interest. In addition, our analyses applied advanced statistical methodology that validly accommodated inclusion of data from multiple cycle outcomes per woman and time-varying exposures and potential confounders. The assessment of early reproductive end points observable only during the IVF process allows for insight into critical points in early pregnancy.

The HCB levels in the study population were close to but generally lower than those of the adult female U.S. population (CDC 2009). Additionally, levels of DDT/DDE observed within Q4 among the women in our study population were lower than those of the general populations of many Asian and African countries where DDT is still in use (CDC 2009; Perry et al. 2006; Rusiecki et al. 2005). Therefore, relations observed within our study population may be estimating the association of these OCs only within the lower tail of the international exposure distribution. Further studies in the general U.S. population and in countries with high pesticide burdens are needed.

## Conclusion

We found significantly greater odds of implantation failure in relation to serum HCB levels. Persistent organic chemicals, despite banned use within the United States, may have an adverse impact on conception and pregnancy progression. Couple-based exposure assessment and study of the adverse effects on gametes, endometrium, and embryo development are needed so that these risks can be better understood.
